# Non-Contact Hand Movement Analysis for Optimal Configuration of Smart Sensors to Capture Parkinson’s Disease Hand Tremor

**DOI:** 10.3390/s22124613

**Published:** 2022-06-18

**Authors:** Prashanna Khwaounjoo, Gurleen Singh, Sophie Grenfell, Burak Özsoy, Michael R. MacAskill, Tim J. Anderson, Yusuf O. Çakmak

**Affiliations:** 1 Department of Anatomy, School of Biomedical Sciences, University of Otago, Dunedin 9016, New Zealand; prash.khwaounjoo@otago.ac.nz (P.K.); gsin255@aucklanduni.ac.nz (G.S.); 2 Medical Technologies Centre of Research Excellence, Auckland 1142, New Zealand; 3 New Zealand Brain Research Institute, Christchurch 8011, New Zealand; sophie.grenfell@nzbri.org (S.G.); michael.macaskill@nzbri.org (M.R.M.); tim.anderson@cdhb.health.nz (T.J.A.); 4 Global Dynamic Systems (GDS) ARGE, Teknopark Istanbul, Istanbul 34906, Turkey; burakozsoy@gmail.com; 5 Department of Medicine, University of Otago, Christchurch 8140, New Zealand; 6 Centre for Health Systems and Technology, Dunedin 9054, New Zealand; 7 Brain Health Research Centre, Dunedin 9054, New Zealand; 8 Centre for Bioengineering and Nanotechnology, University of Otago, Dunedin 9054, New Zealand

**Keywords:** wearable sensors, postural tremor, smart devices, Parkinson’s disease, tremor detection

## Abstract

Parkinson’s disease affects millions worldwide with a large rise in expected burden over the coming decades. More easily accessible tools and techniques to diagnose and monitor Parkinson’s disease can improve the quality of life of patients. With the advent of new wearable technologies such as smart rings and watches, this is within reach. However, it is unclear what method for these new technologies may provide the best opportunity to capture the patient-specific severity. This study investigates which locations on the hand can be used to capture and monitor maximal movement/tremor severity. Using a Leap Motion device and custom-made software the volume, velocity, acceleration, and frequency of Parkinson’s (*n* = 55, all right-handed, majority right-sided onset) patients’ hand locations (25 joints inclusive of all fingers/thumb and the wrist) were captured simultaneously. Distal locations of the right hand, i.e., the ends of fingers and the wrist showed significant trends (*p* < 0.05) towards having the largest movement velocities and accelerations. The right hand, compared with the left hand, showed significantly greater volumes, velocities, and accelerations (*p* < 0.01). Supplementary analysis showed that the volumes, acceleration, and velocities had significant correlations (*p* < 0.001) with clinical MDS-UPDRS scores, indicating the potential suitability of using these metrics for monitoring disease progression. Maximal movements at the distal hand and wrist area indicate that these locations are best suited to capture hand tremor movements and monitor Parkinson’s disease.

## 1. Introduction

Parkinson’s disease (PD) affects 1 to 2 people per 1000 of the population at any one time [1]. PD is a progressive neurodegenerative disease [2] characterised by both motor [3] and non-motor [4] symptoms. Motor symptoms include tremors, bradykinesia, rigidity, and postural instability [5,6] while non-motor symptoms consist of hyposmia, sleep disorders, depression, and olfactory issues [4,7]. These signs and symptoms hinder the performance of their daily activities, reducing their level of independence. At present, there is no curative treatment for PD; rather, treatments are focused on the symptoms [8]. With the aging population, the number of people with Parkinson’s disease is also increasing [6]. With this in mind, it has become more important to easily acquire objective symptom data to monitor and diagnose PD.

Currently, tremors are one of the most identifiable features of PD. Tremor conditions are commonly measured visually in either hospitals or home healthcare environments without specialised equipment [8]. The severity of symptoms, i.e., tremor magnitude is usually assessed by a clinical scoring system: Movement Disorder Society-Sponsored Revision of the Unified Parkinson’s Disease Rating Scale (MDS-UPDRS). Some MDS-UPDRS sub-scores are subjective and rely on visual observations from physicians. These scores, in turn, are sometimes used for determining medical therapies and drug dosages and in clinical trials [9,10]. Furthermore, clinical assessments cannot be performed regularly or continuously in a daily life environment [11]. To enable more frequent and objective ratings of symptoms and disease courses, continuous monitoring systems are being investigated for PD.

With new technologies in healthcare, medical devices/sensors are increasingly utilised as objective methods for diagnosis/monitoring. Wearable sensors are portable, cost-effective, power-efficient, and provide accurate data [12,13]. To date, wearable sensors have shown promise in PD [14,15] and in particular for detecting tremors [16], freezing of gait [17,18], bradykinesia [19,20], and dyskinesia [21]. Commercial health designs of wearable hand devices (similar to rings/watches) are also available such as Kinetigraph and Kinesia^TM^ [22]. These systems perform objective tremor quantification by analysing data obtained from accelerometers or gyroscopes through different computational methods [23]. Recently, several monitoring systems have been developed and tested and have shown promising results for quantifying PD symptoms [21,24].

However, within these studies, the subject’s hand shape is restricted by having to either hold or wear a large measuring device. In addition, the optimal area (i.e., wrist, or which finger joint) to place these devices to capture tremor movements regardless of disease severity is unknown. Identifying the locations of maximal tremor motion allows patients with smaller tremor movements (which potentially can identify subtle symptoms or early signs of PD) to be captured and positions to be defined so comparisons can be standardised. With devices such as smart rings and watches becoming more common for health monitoring [15,25], investigation of the best locations to capture hand tremor movements is needed. Users also have variable preferences as to which location to place a device, i.e., which finger to wear a smart ring. This study makes use of an already available Leap Motion camera to identify and measure movements of multiple joints within the hand simultaneously, including the wrist, to determine locations of maximal tremor movements. The results of the study provide useful initial information for the placement of smart devices for PD monitoring.

## 2. Methods

This study was approved by Health and Disability Ethics Committees of the New Zealand Ministry of Health (URB/09/08/037/AM23) and performed following relevant guidelines and regulations. All participants provided signed consent. Subjects were recruited from the New Zealand Brain Research Institute’s research volunteer database of PD patients. Table 1 shows the demographic distribution and patient characteristics. A total of 55 patients (17 female, 38 males, mean age ± SD = 71.7 ± 6.54) who were diagnosed by a neurologist with PD participated in the experiment. All patients were under dopaminergic replacement treatment, and their disease duration was 11.1 ± 4.8 years. In total, 20 left-sided onsets, 34 right-sided onsets, and 1 with bilateral onset were included. All of the participants were right-handed. Trained clinical personnel evaluated MDS-UPDRS (approval granted) scores bilaterally within a day of any data acquisition. These scores encompassed parts I (non-motor), II (motor experience daily living), and III (motor examination) which includes questions 3_15a and 3_15b relating to the postural tremor severity for the right and left hand, respectively.

### 2.1. Inclusion Criteria

The inclusion criteria for the study were individuals who were between the ages of 50 and 90 years and who have been diagnosed with at least stage two on the Hoehn and Yahr scale of Parkinson’s Disease severity [26].

### 2.2. Exclusion Criteria

Individuals that exhibited musculoskeletal or other neurological conditions [27] which may affect movement were excluded from the study. Significant dyskinesia was observed in two patients; thus, these patients were removed from the study. Individuals presenting with orthopaedic disorders, individuals that use orthotic devices, or those with artificial joints were further excluded from the study.

### 2.3. Data Collection and Measurement

A Leap Motion Controller (LMC), (Leap Motion, Inc., San Francisco, CA, USA) infrared camera-based motion controller device was used to capture the postural tremors. The LMC is a cost-effective device that is able to capture numerous hand joint positions and movements simultaneously. The device has also been used extensively to measure movements related to Parkinson’s disease, such as postural tremors [28,29,30,31], and to estimate hand dexterity UPDRS scores in PD patients [32]. Weichert et al. [33] analysed the accuracy of the Leap Motion Controller and found that it can achieve 0.7 mm overall average accuracy in all 3 axes. This result is comparable to the average human hand accuracy of 0.4 mm.

**Figure 1:**
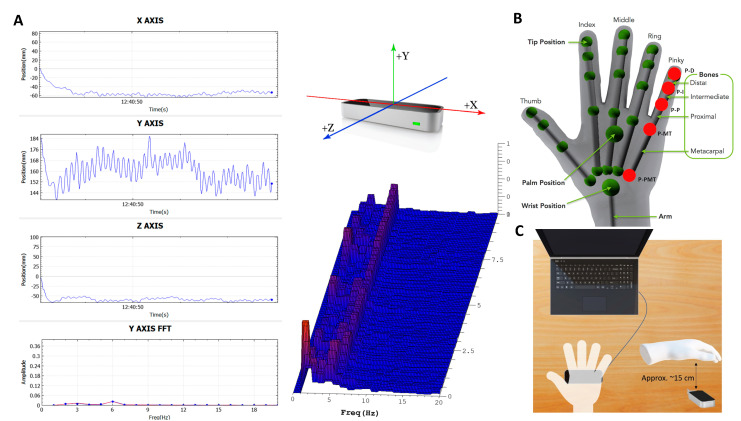
Hand joint acquisition pipeline: (**A**) custom-made application’s layout for acquisition of Leap Motion hand data (x, y, z axes), middle top: orientation of Leap Motion x, y, z coordinates and middle bottom: frequency profile (red indicates high frequency magnitude) (**B**) hand joint locations; red dot provides example locations for pinky finger (modified from Leap Motion). Letters next to red dots show the labelling conventions, i.e., P-PMT—pinky proximal metacarpal; (**C**) experimental configuration for acquisition of data from the Leap Motion device.

Custom-made software was used to capture tremor movements. The application (Figure 1A shows the application interface) was developed on top of the Leap Motion SDK to capture all positions simultaneously from all the fingers (thumb—T, index—I, middle—M, ring—R, and pinky—P, i.e., little finger) and respective joints (distal—D, intermediate—I, proximal—P, metacarpal—M, proximal metacarpal—PMT), including the wrist, (Figure 1B). Figure 1B also shows the naming convention example for the pinky finger. Within the Leap system, the thumb uses the same bones as the other fingers, although an anatomical thumb has no intermediate phalange. To compensate, the Leap Motion model and naming system of the thumb has a zero-length metacarpal bone (Leap Motion API) i.e., the Leap system intermediate and proximal thumb phalanges are analogous to the anatomical proximal phalange and metacarpal thumb bones respectively.

Each patient was asked to hold their hand (right and left separately), over the Leap Motion device for 10 s. During the recordings, patients were asked to hold each hand flat over the Leap with fingers apart from each other (Figure 1C). Each hand was recorded three times and any analysis output was subsequently averaged.

### 2.4. Data Analysis

#### 2.4.1. Volumes

All data analysis was conducted using MATLAB 2020a (MathWorks, Natick, MA, USA). The average sampling rate of the Leap Motion Controller during the study was approximately 100 Hz; to account for the variable acquisition rate, the change in the position data was interpolated with a cubic spine and resampled at a frequency of half of the average sampling rate, i.e., 50 Hz. The magnitudes of the positional data from the Leap Motion Controller were then filtered using a 4th-order band-pass Butterworth Filter. The Butterworth filter that was used had a low-pass cut-off frequency of 20 Hz and a high-pass cut-off of 2.5 Hz to eliminate frequencies that were not of interest, such as those due to physiological factors such as breathing and heart rate [34].

**Figure 2:**
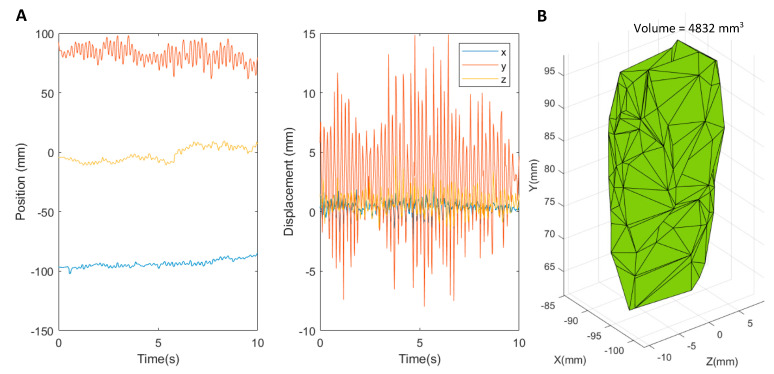
Example Leap Motion tremor measurements of right index finger distal joint (I.D): (**A**) x, y, and z positional information from the Leap over 10 s; (**B**) tremor volume calculated from the three-dimensional positional information.

The filtered x, y, and z data (Figure 2A) were then used to create a volume of the resultant movement. The MATLAB function *alphaShape* was utilised to create a representation of the movement. This function uses a set of points in 3D and Delaunay triangulation to create a tetrahedral mesh. This mesh is estimated by creating a bounding polyhedron that envelopes the 3D location of the joint movement (Figure 2B).

#### 2.4.2. Tremor Metrics

**Figure 3:**
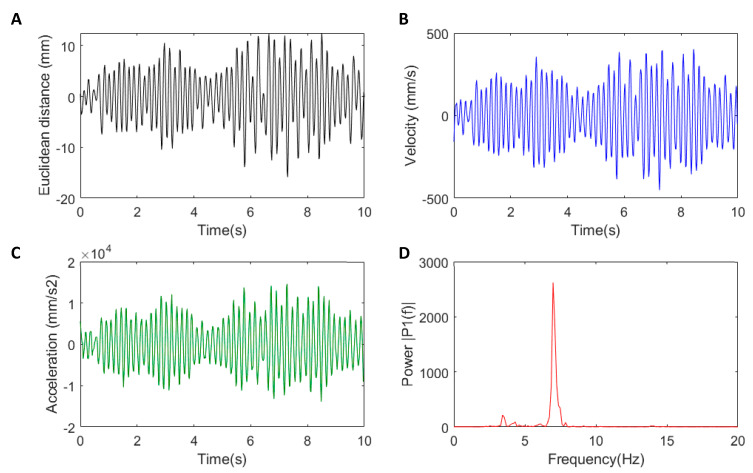
Leap Motion feature extraction from right-hand index finger distal: (**A**) Euclidean distance calculated from x, y, z position; (**B**) velocity of movement in mm/s (blue); (**C**) acceleration of movement in mm/s^2^ (green); (**D**) peak frequency of the acceleration ~7.1 Hz (red). Colours indicate extracted metric, same colour scheme used in subsequent figures.

Euclidean distance (Equation (1)) was calculated from the three-dimensional sampled positions. The origin of the coordinate system of the Leap Motion Controller is centred at the top and middle of the surface of the Leap and is assumed to remain stationary. The change in Euclidean displacement (Equation (2) and Figure 3A) was then calculated for each recorded frame to determine the magnitude of the change in distance with the new associated time (Equation (3)). This displacement metric considers the magnitude of movement from each axis. This approach also standardises the data as a normalisation process [35].

(1) $$\;d_{n}=\sqrt{x_{n}^{2}+y_{n}^{2}+z_{n}^{2}}$$

(2) $$\;D_{n}=d_{n+1}-d_{n}$$

(3) $$\;T_{n}=t_{n+1}$$

where

*n* is the frame number;

(*x_n_*, *y_n_*, *z_n_*) are the positional coordinates associated with frame *n*;

*d_n_* is the Euclidean distance;

*d_n+_*_1_ is the Euclidean distance associated with frame *n* + 1;

*D_n_* is the new Euclidean displacement associated with frame *n*;

*t_n+_*_1_ is the time associated with frame *n* + 1;

*T_n_* is the new time associated with *D_n_*.

The displacements enabled the commonly used metrics of velocity and acceleration of the tremor movement to be determined. A 4th-order Savitzky–Golay FIR filter, with a frame size of 11 points, was implemented in MATLAB using the *sgolay* function to smooth and perform a 1st-order and 2nd-order differentiation on the Euclidean displacement. This allowed for an approximation of the change in magnitude of the velocity (Figure 3B) and acceleration (Figure 3C) while removing higher frequency noise that was amplified from differentiation. Finally, a fast Fourier transform of the acceleration was used to compute power spectral density and peak (dominant) tremor frequency (Figure 3D). For inter-joint comparisons, root-mean-squared (RMS) values were calculated for the velocities and acceleration of tremors. All features were grouped by the subject and signal and were subsequently averaged, resulting in a unique scalar combining the magnitude of the three recordings, thus making the feature values independent of any inter-trial variance in the statistical analysis.

#### 2.4.3. Statistical Analyses

Prism (Version 9.2.0) Statistical software (GraphPad Inc., La Jolla, CA, USA) was used. The tremor metrics were tested for normality using Shapiro–Wilk tests. Log transformation was subsequently applied for features prior to running mixed two-way repeated-measures analyses of variance. Geisser–Greenhouse correction was also applied, and Tukey Post hoc comparisons were used to determine significant differences between the hands and/or joints (*p* < 0.05; *n* = 55). As a supplementary analysis correlations between calculated measures and MDS-UPDRS scores, questions 3_15a/b, parts I, II, and III were determined using Spearman’s correlation coefficients.

## 3. Results

**Figure 4:**
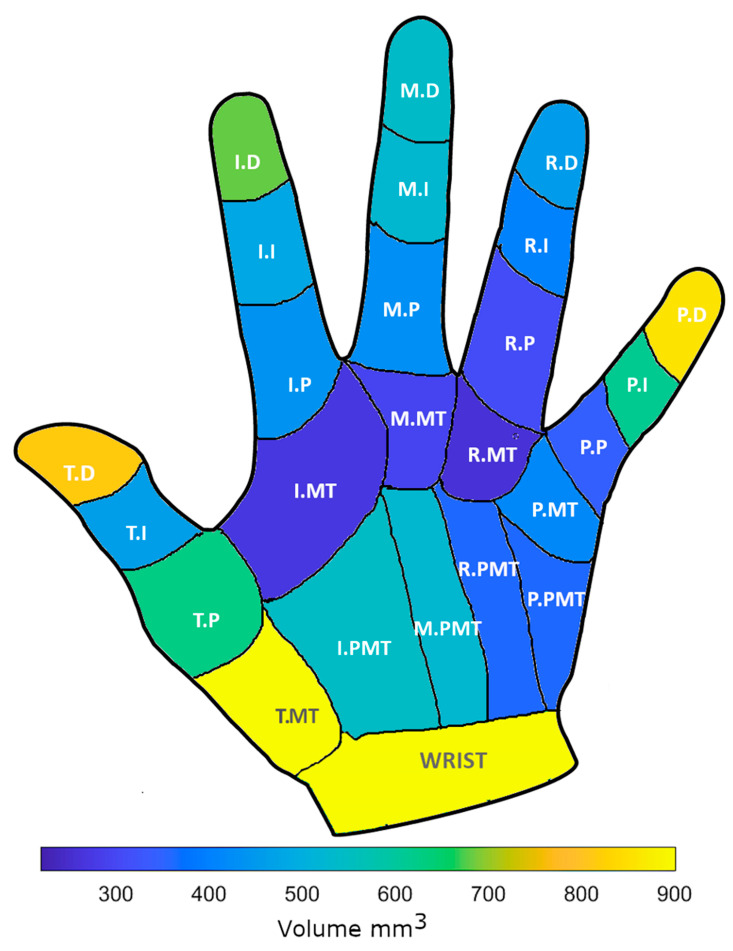
Example right-hand volume and localisation of the joints, e.g., T.D is thumb distal. Colour indicates the magnitude of the volumes. Note: the thumb intermediate phalange (T.I) is a result of the standard naming system of the Leap Motion model. The Leap system intermediate and proximal thumb phalanges are analogous to the anatomical proximal phalange and metacarpal thumb bones respectively.

**Figure 5:**
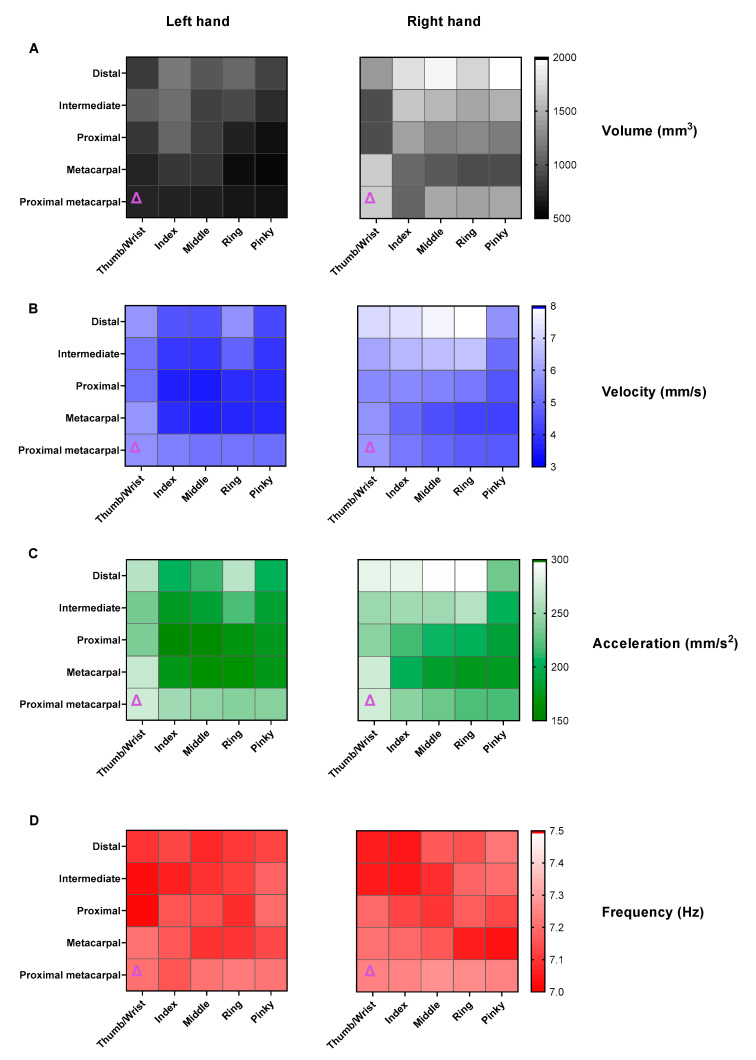
Right- and left-hand joints mean Leap Motion metric values. Lighter shade of colour indicates greater magnitudes. Δ represents the wrist: (**A**) tremor volumes (mm^3^); (**B**) root-mean-squared (RMS) velocity (mm/s); (**C**) RMS acceleration (mm/s^2^); (**D**) peak frequency (Hz).

Figure 4 displays an example of the tremor volumes for a right hand and the naming convention for the joints. In this example, the distal locations along each finger have the highest movement volumes. Note: the thumb intermediate phalange (T.I) is a result of the standard naming system of the Leap Motion model. The Leap system intermediate and proximal thumb phalanges are analogous to the anatomical proximal phalange and metacarpal thumb bones respectively. Figure 5A shows the average movement volumes of the hand joints for all participants. For both right and left hands, the volume of movement for each finger increased from metacarpal to distal locations, there were, however, no significant differences between all distal finger locations of the hand, including the wrist and the rest of the joints.

For the velocities and acceleration, the distal locations trended towards being the highest for each finger for the right hand, while on the left hand, the highest values were observed in the proximal metacarpal (PMT) locations and wrist (Figure 5B,C).

For the right hand, the wrist had significantly higher velocities than the majority of hand locations except for the PMT for the middle, ring, index fingers, and all thumb locations (Table 2). The index, ring, and middle distal locations were also not significantly different. The acceleration values showed similar trends—the wrist accelerations were significantly higher for all locations except the distal locations of the index, middle, ring pinky fingers, and all thumb locations (Table 2). Conversely, for the left hand, the wrist had significantly higher velocities than all joints on each finger except the distal PMT/all thumb locations and significantly higher accelerations than the ring distal and all thumb locations (Table 2). Frequencies showed no significant difference between any of the hand join locations for both hands (Figure 5D).

When comparing the hands, the right hand on average showed greater tremor volumes (*p* = 0.0001). The left hand also had significantly lower velocity (*p* = 0.0001) and acceleration (*p* = 0.008) than the right hand; however, there was no significant difference in frequencies.

**Figure 6:**
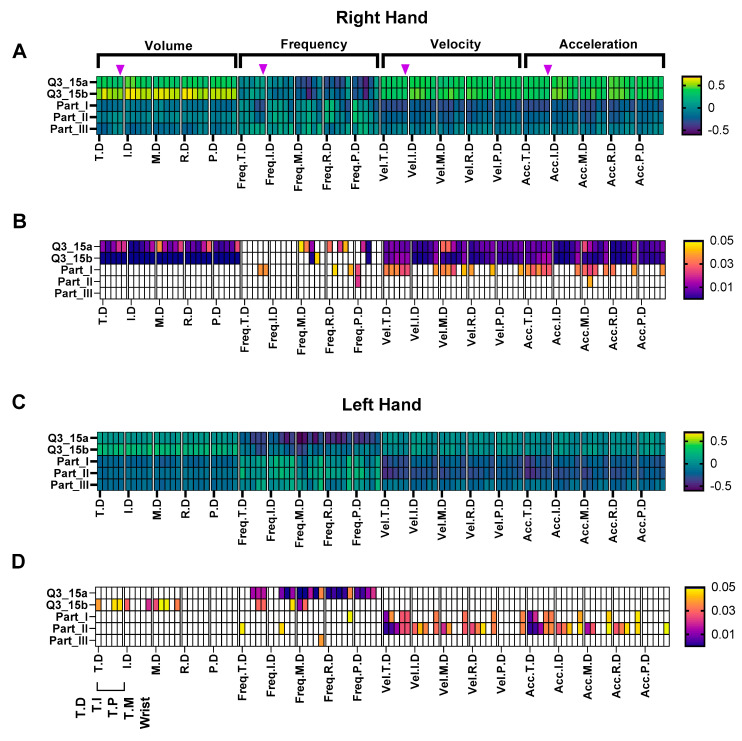
Hand movement metrics correlations with MDS-UPDRS scores (questions 3_15a/b, parts I, II, III). Each set of five squares following on from the label represents locations on each finger/thumb (e.g., T.D, T.I, T.P, T.MT, wrist): (**A**) right-hand MDS-UPDRS score correlations. Purple arrow head ▼ represents the wrist; (**B**) right-hand *p* values, white squares represent non-significance (*p* > 0.05); (**C**) left-hand MDS-UPDRS score correlations; (**D**) left-hand *p* values, white squares represent non-significance (*p* > 0.05).

As a supplementary analysis, the features of the Leap Motion were compared with the MDS-UPDRS scores. The volume, frequency, and velocities significantly correlated with the MDS-UPDRS scores and specifically questions 3_15a and 3_15b which are related to the postural tremor severity of the right and left hand, respectively. The results show that the right-hand volumes, velocities, and accelerations for all joints correlated significantly with MDS-UPDRS (question 3_15a/b) values associated with the postural tremor (Figure 6A). In addition, the frequencies of the movements were found to significantly correlate with the middle, ring, and pinky fingers with the postural tremor MDS-UPDRS ratings (Figure 6A). MDS-UPDRS part I scores also showed significant correlations with a number the hand movement velocities/accelerations.

Significant left-hand correlations were observed between the tremor volumes for the thumb, index, middle fingers, and MDS-UPDRS Q3_15b (left-hand postural tremor severity) and not the other velocities and accelerations (Figure 6B). All left-hand correlations were not as high as those of the right hand. In contrast to the right hand, several left-hand joint velocities/accelerations displayed a significant correlation with part II scores, and the frequency of the majority of joints showed significant correlations to Q3_15a. No significant correlations were observed with part III for either hand.

## 4. Discussion

Wearable devices are increasingly being used for health monitoring. Research is required to identify the optimal body locations of such devices to monitor specific conditions. Parkinson’s disease (PD) is a clinical condition incorporating technology/devices, such as rings and watches, for monitoring disease progression [12,15,25]. For objective quantification of PD severity, it is important to determine the best locations of these devices for capturing the subtleties of the symptoms. Multiple methods have been used to investigate the severity of PD using accelerometers [12,15,36] and have been able to differentiate between PD and non-PD patients [37,38]. To the best of our knowledge, these investigations have not compared different regions within the hand, including the wrist and finger joints, to find maximal tremor locations. This paper presents the first study of its kind to investigate the localisation of PD movements of multiple hand joints, including all fingers and the wrist, to find the optimal/standard location to capture maximal tremor movements. Identification of these locations will enable smaller tremors to be acquired, whereas placement at other locations may result in non-identification of tremor movements. Tremor volumes were also extracted, and a supplementary initial correlation was conducted to investigate their utility for predicting PD severity. The results identified the best joint locations to detect maximal movements/tremors to be the wrist and distal finger joints and also provided preliminary validation of each location as an area that can be potentially used to monitor PD.

The distal joints of the fingers and the wrist trended towards having the highest magnitude of volumes, velocity, and acceleration, wherein values of acceleration and velocity agree with the previous literature [39]. On the left hand, the wrist was found to have significantly greater velocity and acceleration than all joints except for proximal metacarpal regions and ring distal joint, respectively. For the right hand, the wrist movement was found to be significantly greater in velocity and acceleration than the metacarpal joints of all fingers, where rings are normally worn, however, no significant differences were observed for the distal locations. Greater distal magnitudes are to be expected, as the distal locations have the highest degrees of freedom; this is also in line with the wrist which has a high degree of mobility. These findings indicate that the best locations to capture the maximal movement on the right hand are the distal locations of the hand or at the wrist. This validates the locations used for devices such as Kinesia One^TM^ [22], normally placed distally on the index finger, and also the use of watch devices such as the PKG watch [40].

Other variables such as the frequency of tremors did not vary significantly between the joints, showing that the observable changes related to tremors are likely to be better detected by the magnitude of the movement. This is in line with MDS-UPDRS ratings, where amplitudes of movement are identified [41]. Past studies have also shown that postural tremors tend to vibrate at a comparable frequency range (5–9 Hz) to that of this study [42,43,44,45]. The average values of ~7 Hz are slightly higher than that seen in the literature, but this is likely attributed to the utilisation of Euclidean distance acceleration. This form of acceleration takes into account all three axes to determine frequency response; hence, the additive nature of the frequencies is likely to increase the acquired peak frequencies.

Additionally, when the left and right hands were compared, the right hand showed significantly greater values for all metrics. All patients in this study were right-handed, and approximately 60% of the patient’s side of onset was on the right side. Previous studies in the literature state that the dominant side of symptoms often occurs with handedness [37], and patients with left-dominant symptoms have less severe patterns of motor responses [46]. Results from this study indicated for the predominantly right-handed and right side of onset PD patients, the right hand i.e., the ipsilateral side is potentially the best location to detect maximal movements. However, further testing and analysis with more patients that are left-handed/left-sided onset are needed to confirm this finding.

A supplementary investigation to provide initial validation of the use of the multiple joint locations’ volumes, velocities, and accelerations features was conducted using MDS- UPDRS score correlations. Consistent significant correlations with the all-hand joint volumes, velocities, and accelerations and MDS-UPDRS scores were observed with the right hand. Fewer significant correlations for the left hand were found, and this is likely due to the relatively lower magnitudes in metrics as a result of 60% of the patient’s right side of onset and factors mentioned previously. This, in turn, may have reduced the separation of tremor severity for the left hand and, consequently, resulted in lower correlations. Further investigations with more left-handed/left side of onset patients are required to explore this relationship. The volume features extracted from the Leap Motion also showed significant correlations to the MDS-UPDRS scores for all locations on the right hand (Q3_15a) and a number of volumes of thumb, index, and middle for Q3_15b of the left hand. While MDS-UPDRS scores are limited to a five-point Likert-type scale (ranging from 0 to 4), utilising a combination of the movement metrics such as volumes, accelerations may be able to provide greater resolution (i.e., due to the continuous nature of volumes/accelerations) in monitoring the severity of the disease. With further investigation, the Leap Motion device and the outputs highlighted in this study can be used to provide greater insight into the disease severity which, in turn, will allow more precise drug dosages. These may enable the effectiveness of PD drugs such as levodopa to be optimised and fine-tuned which, in turn, may reduce and delay the tendency to motor fluctuations [9,11].

## 5. Conclusions

The outcome of this study indicated that smart devices are best placed on either the wrist or more distal locations of the hand to capture maximal tremor movements. In addition, these devices should be placed on the ipsilateral side i.e., right side if the patient is right-handed and has a right side of onset. These findings showed that, in the future, everyday smartwatches with accelerometers can be implemented with software to detect the maximal movement of the hands during tremors. Additionally, for smart rings to monitor PD tremor motion to a high level of sensitivity, they may need attachments that allow accelerometers to be extended to the distal locations. The main advantages of using smart devices for tremor recording and analysis are their non-invasiveness and ease of use, which could translate into routine clinical or even community monitoring. Furthermore, preliminary investigations on the use of the Leap Motion system and features such as tremor volumes show promise as a tool for obtaining the severity of tremors. To build on the findings of this research, a longitudinal study utilising remote transmission of data from the home environment is needed to fully determine the utility of the use of such devices and locations.

## Figures and Tables

**Table 1:** Demographics and disease characteristics (mean ± standard deviation (range)) of the participants.

Characteristics	Participants with Parkinson’s Disease
*n* (male)	55 (38)
side of onset (left, right, bilateral)	20, 34, 1
age (years)	72 ± 7 (53–87)
Hoehn and Yahr (1–5)	2.0 ± 0.5 (2–3)
Levodopa equivalent dose (mg)	1050 ± 586 (155–2900)
Disease duration (years)	11 ± 5 (3–27)
MDS-UPDRS I	10 ± 5 (2–24)
MDS-UPDRS II	11 ± 6 (1–29)
MDS-UPDRS III	34 ± 12 (14–61)
Q3_15a (Postural Tremor Right hand)	1 ± 0.5 (0–3)
Q3_15b (Postural Tremor Left hand)	1 ± 0.5 (0–3)

**Table 2:** Wrist vs. hand joint comparisons for log-transformed velocity and acceleration. * *p* value < 0.05, ** < 0.01, *** < 0.001.

Wrist Compared to	Velocities				Acceleration			
	LH		RH		LH		RH	
T.D	ns	>0.999	ns	>0.999	ns	>0.999	ns	>0.999
T.I	ns	>0.999	ns	>0.999	ns	>0.999	ns	>0.999
T.P	ns	>0.999	ns	>0.999	ns	>0.999	ns	>0.999
T.MT	ns	>0.999	ns	>0.999	ns	>0.999	ns	>0.999
I.D	***	<0.001	ns	0.147	***	<0.001	ns	0.106
I.I	***	<0.001	***	<0.001	***	<0.001	***	<0.001
I.P	***	<0.001	***	<0.001	***	<0.001	***	<0.001
I.MT	***	<0.001	***	<0.001	***	<0.001	***	<0.001
I.PMT	ns	0.999	ns	0.969	***	<0.001	***	<0.001
M.D	***	<0.001	ns	0.723	***	<0.001	ns	0.963
M.I	***	<0.001	***	<0.001	***	<0.001	*	0.014
M.P	***	<0.001	***	<0.001	***	<0.001	***	<0.001
M.MT	***	<0.001	***	<0.001	***	<0.001	***	<0.001
M.PMT	ns	0.937	ns	0.380	***	<0.001	***	<0.001
R.D	*	0.026	ns	>0.999	ns	0.238	ns	>0.999
R.I	***	<0.001	**	0.005	***	<0.001	ns	0.117
R.P	***	<0.001	***	<0.001	***	<0.001	***	<0.001
R.MT	***	<0.001	***	<0.001	***	<0.001	***	<0.001
R.PMT	ns	0.782	ns	0.067	***	<0.001	***	<0.001
P.D	***	<0.001	*	0.010	***	<0.001	ns	0.201
P.I	***	<0.001	***	<0.001	***	<0.001	**	0.002
P.P	***	<0.001	***	<0.001	***	<0.001	***	<0.001
P.MT	***	<0.001	***	<0.001	***	<0.001	***	<0.001
P.PMT	ns	0.894	*	0.041	***	<0.001	***	<0.001

## Data Availability

The data presented in this study are available on request from the corresponding author. The data are not publicly available due to the clinical nature of the data.
